# MAG2 and MAL Regulate Vesicle Trafficking and Auxin Homeostasis With Functional Redundancy

**DOI:** 10.3389/fpls.2022.849532

**Published:** 2022-03-16

**Authors:** Xiaohui Ma, Xiaonan Zhao, Hailong Zhang, Yiming Zhang, Shanwen Sun, Ying Li, Zhengbiao Long, Yuqi Liu, Xiaomeng Zhang, Rongxia Li, Li Tan, Lixi Jiang, Jian-Kang Zhu, Lixin Li

**Affiliations:** ^1^Key Laboratory of Saline-Alkali Vegetation Ecology Restoration, College of Life Sciences, Ministry of Education, Northeast Forestry University, Harbin, China; ^2^Institute of Crop Science, Zhejiang University, Hangzhou, China; ^3^Shanghai Center for Plant Stress Biology, Center of Excellence in Molecular Plant Sciences, Chinese Academy of Sciences, Shanghai, China

**Keywords:** MAG2 and MAL, vesicle trafficking, auxin homeostasis, plant development and stress response, proteomic analysis

## Abstract

Auxin is a central phytohormone and controls almost all aspects of plant development and stress response. Auxin homeostasis is coordinately regulated by biosynthesis, catabolism, transport, conjugation, and deposition. Endoplasmic reticulum (ER)-localized MAIGO2 (MAG2) complex mediates tethering of arriving vesicles to the ER membrane, and it is crucial for ER export trafficking. Despite important regulatory roles of MAG2 in vesicle trafficking, the *mag2* mutant had mild developmental abnormalities. MAG2 has one homolog protein, MAG2-Like (MAL), and the *mal-1* mutant also had slight developmental phenotypes. In order to investigate MAG2 and MAL regulatory function in plant development, we generated the *mag2-1 mal-1* double mutant. As expected, the double mutant exhibited serious developmental defects and more alteration in stress response compared with single mutants and wild type. Proteomic analysis revealed that signaling, metabolism, and stress response in *mag2-1 mal-1* were affected, especially membrane trafficking and auxin biosynthesis, signaling, and transport. Biochemical and cell biological analysis indicated that the *mag2-1 mal-1* double mutant had more serious defects in vesicle transport than the *mag2-1* and *mal-1* single mutants. The auxin distribution and abundance of auxin transporters were altered significantly in the *mag2-1* and *mal-1* single mutants and *mag2-1 mal-1* double mutant. Our findings suggest that MAG2 and MAL regulate plant development and auxin homeostasis by controlling membrane trafficking, with functional redundancy.

## Introduction

Auxin is a central phytohormone for almost all aspects of plant growth and development (reviewed in Gomes and Scortecci, 2021), and response to environmental stimuli (reviewed by Zhao, 2018). Auxin homeostasis regulated by coordination of auxin biosynthesis, catabolism, transport, conjugation, and deposition optimizes plant development and adaption to environmental stress (Bhalerao and Bennett, 2003; Blakeslee et al., 2019). Auxin gradients determine developmental outcomes (Leyser, 2005; Habets and Offringa, 2014; Zhao, 2018). Both roots and shoots exhibit auxin gradients across longitudinal axes, and auxin levels are generally most concentrated in organ meristems and rapidly dividing tissues (Kramer and Bennett, 2006). Auxin transport is controlled mainly by AUXIN1 (AUX1), PIN-FORMED (PIN), and PIN-LIKES (PILS) family carriers. These proteins coordinately control auxin intercellular and intracellular transport and determine plant morphogenesis (Mravec et al., 2009; Barbez et al., 2012). Canonical PIN proteins such as AtPIN1-4 and AtPIN7 localize in the plasma membrane (PM) asymmetrically and play an overarching role in plant development by regulating directional cell-to-cell auxin transport (reviewed by Naramoto, 2017; Béziat and Kleine-Vehn, 2018). PILS proteins are observed to localize only in the endoplasmic reticulum (ER) (Barbez et al., 2012; Sauer and Kleine-Vehn, 2019), while, non-canonical PINs display diverse localization. For instance, AtPIN5 exhibits cell type-dependent localization, at the PM in aerial tissues and intracellular localization in root vascular cells (Ganguly et al., 2014); AtPIN6 shows dual localization in the ER and the PM (Simon et al., 2016; Ditengou et al., 2018); PIN8 is colocalized with PIN5 in the ER in pollen (Ding et al., 2012). Non-canonical PIN and PILS proteins likely sequester auxin in the ER and have an impact on cellular auxin signaling and homeostasis (Mravec et al., 2009; Barbez et al., 2012; Béziat et al., 2017; Middleton et al., 2018; Feraru et al., 2019; Sun et al., 2020).

After being synthesized and assembled in the ER (Borgese et al., 2006), canonical PIN proteins are delivered to the PM through the secretory pathway, and they maintain their homeostasis in the PM by the cycling machinery (Naramoto, 2017). Phosphorylation of PIN proteins, which appears to control both PIN directional delivery and activities, is regulated by kinases, D6 protein kinases (D6PKs), PINOID (PID), wavy root growth (WAG)1, WAG2, and protein phosphatese 2A (PP2As) (Friml et al., 2004; Michniewicz et al., 2007; Dhonukshe et al., 2010; Zourelidou et al., 2014; Weller et al., 2017; Barbosa et al., 2018; Zhou and Luo, 2018). The impact of PID and PP2As on PIN phosphorylation status determines PIN cycling and maintains PIN polar localization (Máthé et al., 2021).

The vesicle trafficking system maintains organelle identities and homeostasis to contribute to proper cellular activities. Recognition machineries of a donor with target membranes consist of tethering factors, Ras-related in brain (RABs), ADP-ribosylation factors (ARFs), guanine nucleotide exchange factors (GEFs), etc. (Lamber et al., 2019; Homma et al., 2021). Tethering factors mediate the first contact between arriving vesicles and target membrane (Grosshans et al., 2006), and transfer the machinery to downstream factors such as soluble *N*-ethylmalemide sensitive factor attachment protein receptors (SNAREs) (Wang et al., 2017). SNAREs facilitate membrane fusion of transport vesicles with target membranes. According to sequences of center amino acids in the SNARE motif, SNARE proteins are classified into Q-SANREs (including Qa-, Qb-, and Qc-SNAREs) and R-SNAREs. Specific combination of R- with Q-SNAREs forms a SNARE complex to drive membrane fusion (Fasshauer et al., 1998).

Tethering factors could be divided into two classes: long single coiled-coil proteins such as MAG4/Atp115 (Whyte and Munro, 2002; Takahashi et al., 2010), and multisubunit complexes (Bröcker et al., 2010; Vukašinović and Žárský, 2016; Ravikumar et al., 2017; Zhao et al., 2018). Different tethering factors localize in distinct compartments as specific recognition machineries (Vukašinović and Žárský, 2016; Ravikumar et al., 2017). For example, the exocyst complex mediates tethering of post-Golgi vesicles to the PM (Saeed et al., 2019). The yeast Dsl1 complex consisting of Dsl1p, Sec39p, and Tip20p is localized in the ER and regulates Golgi-to-ER retrograde transport (Andag and Schmitt, 2003; Ren et al., 2009). The downstream SNAREs are Use1p, Sec20p, and Ufe1p (Linders et al., 2019). Our previous study has demonstrated that the *Arabidopsis* homolog complex of the Dsl1 complex is the MAG2-MIP1-MIP2-MIP3 complex (Li et al., 2006, 2013; Zhao et al., 2018). The MAIGO2 (MAG2) complex cooperates with ER-localized SANRE complex components, Qa-AtSYP81 and Qc-AtSec20, and potentially regulates Golgi-to-ER vesicle trafficking (Li et al., 2006, 2013). The *mag2* and *mip1/2/3* mutants abnormally accumulated precursors of seed storage proteins (SSPs, e.g., 2S albumins and 12S globulins) inside the ER lumen in seed cells (Li et al., 2006, 2013). In addition to important regulatory roles in membrane trafficking, MAG2 and MAG2-interacting proteins (MIPs) are also involved in response to abiotic stress and hormone, such as salinity, heat shock and osmotic stress, and abscisic acid (ABA) and gibberellic acid (Zhao et al., 2013, 2018; Zhao and Lu, 2014). Despite the important regulatory roles of MAG2 in vesicle transport and stress response, *mag2* mutants just exhibit mild developmental abnormalities. It is reported that MAG2 has a homolog protein, MAG2-like (MAG2-Like (MAL), *At1g08400*) (Zhao et al., 2013; Zhao and Lu, 2014). We isolated a T-DNA insertion mutant, *mal-1*, and found that it also had slight developmental phenotypes. In order to analyze MAG2 and MAL function in plant development, we generated a double mutant, *mag2-1 mal-1*. As expected, the *mag2-1 mal-1* double mutant had serious developmental defects such as decreased germination activities, dwarf and partial seed abortion, and abnormal response to salt and osmotic and ABA stress. SSP precursors also accumulated at a higher level in the double mutant seeds than in the single mutant seeds. Proteomic analysis revealed that signaling, metabolism, and stress response were affected in *mag2-1 mal-1*, especially membrane trafficking, auxin biosynthesis, signaling, and transport. Biochemical and cell biological analysis indicated that the *mag2-1 mal-1* double mutant had more serious defects on vesicle transport than the single mutants. Auxin distribution and auxin transporter accumulation were significantly altered in *mag2-1*, *mal-1*, and *mag2-1 mal-1*. Our findings suggested that MAG2 and MAL regulate plant development, auxin homeostasis, and stress response potentially by controlling vesicle trafficking, and that they are functionally redundant.

## Materials and Methods

### Plant Materials and Growth Conditions

*Arabidopsis thaliana* ecotype Col-0 was used as wild-type plants. The T-DNA-tagged line (*mal-1*, GABI_kat_288E12) was provided by the *Arabidopsis* Biological Resource Center (ABRC) at Ohio State University. The *mag2-1* mutant was from our previous study (Li et al., 2006, 2013; Zhao et al., 2018). Homozygous plants were obtained by PCR screening using gene-specific primers. *Arabidopsis* seeds were surface-sterilized and sown either on soil or in 0.8 or 1.2% agar with 1/2 Murashige and Skoog medium (PhytoTech, China) and 1% (w/v) sucrose. The plants were grown at 22°C under 16: 8-h/light: dark cycles.

Transgenic plants (Col-0 background) of overexpressing TAP-tagged *MAL* were generated using a modified pNTAPa vector described by Li et al. (2006, 2013) and Zhao et al. (2018).

### RNA Extraction and RT-PCR Analysis

Total RNA was isolated using RNAiso Plus (9109; TAKARA, Japan). Total RNA 0.5–1 μg was reverse transcribed using PrimeScript™ RT Master Mix (Perfect Real Time) (RR036A; TAKARA, Japan). Semiquantitative RT-PCR and RT-qPCR were performed according to the manufacturer’s instructions. *ACT2* was used as an endogenous control for RT-PCT.

### Antibodies and Immunoblot Analysis

Sodium dodecyl sulfate polyacrylamide gel electrophoresis (SDS)–PAGE and immunoblot analysis were performed as described previously (Shimada et al., 2003). Antibody dilutions were as follows: anti-BiP (AS09 481; Agriser, Sweden) 1:2,000, anti-12S 1:20,000, anti-2S3P 1:5,000 (Li et al., 2006); anti-myc (9E10:sc-40; Santa Cruz Biotechnology, Inc., Shanghai, China) 1:2,000; anti-TUA (R0267-1a; Abiocode, CA, United States) 1:2,000. The dilution of horseradish peroxidase-conjugated rabbit antibodies raised against rabbit IgG (ZB2301, ZSGB-BIO, Beijing, China) was 1:5,000. Signals were detected using an enhanced chemiluminescence (ECL) detection system (LAS-4000; Fujifilm, Japan).

### Yeast Two-Hybrid Assay

For the yeast two-hybrid assay, *AtSYP81*, *AtSEC20*, and *MAG2* constructs were generated as described in our previous study (Li et al., 2006). The cDNA of *MAL* was amplified and fused in-frame downstream of the GAL4 activation domain in the pGADT7 vector or downstream of the GAL4 DNA binding domain in the pGBKT7 vector. We introduced paired constructs into strain AH109 of *Saccharomyces cerevisiae* (Clontech, United States) and selected on SD/-Leu/-Trp (synthetic defined plate deficient in both Leu and Trp) plates. The interactions were examined on SD/-Leu/-Trp/-His/-Ade plates.

### Preparation of Microsomal Proteins

Fractionation was performed basically as described previously (Li et al., 2006). Two grams of roots from 7-day-old seedlings were harvested and ground to fine powder in liquid nitrogen. Ground tissues were suspended in a homogenization buffer (50 mM Tris–HCl, 2 mM ethylene diamine tetraacetic acid (EDTA), 10 mMβ-mercaptoethanol, 250 mM sucrose, pH 7.5) and centrifuged at 8,000 × *g* for 15 min at 4°C to remove debris. The supernatant was recovered, and we repeated centrifugation. The resulting supernatant was ultracentrifuged (Optima™ L-100 XP Ultracentrifuge; Beckman Coulter, United States) at 100,000 × *g* for 1 h at 4°C. The pellet was surface washed with 80% cold acetone and subjected to proteomics analysis.

### Label-Free Analysis

Label-free analysis was performed as described previously (Sheng et al., 2015), with modifications. The abundance of each protein in multiply samples was normalized by total intensity. Briefly, peptides were harvested by centrifugation, acidified with 1% CF3COOH, and subsequently dried with a refrigerated CentriVap concentrator (Labconco, Kansas, MO, United States). The dried peptide mixture powder from each digested sample was reconstituted with 30 μl 2 mM TEAB buffer (pH 8.5). Prior to mass spectrum (MS) analysis, samples were desalted onto an Empore C18 47-mm disk (3M) (Ishihama et al., 2006). The dried peptides were resuspended in 0.1% (v/v) formic acid solution and then analyzed with a Q Exactive mass spectrometer (Thermo Electron Finnigan, San Jose, CA, United States). The mass spectra were submitted to the Maxquant software (version1.4.1.2) for peptide identification, and searched against *A*. *thaliana* protein sequences (Tair) downloaded in 2014. The following parameters were used: carbamidomethylation of Cys was set as fixed modification, phospholation of STY, oxidation of M, and acetylation of protein N terminal were set as variable modifications, and a maximum of two missed cleavages was allowed. The false discovery rate for peptide, protein, and site identification was set to 1% (Cox et al., 2011). A total of 4,546 proteins were identified in both wild-type and *mag2-1 mal-1*, and 515 were differently accumulated proteins (DAPs) in the *mag2-1 mal-1* double mutant. The DAPs were filtered with change ratio >1.2 or *p* ≤ 0.05, and 124 of the DAPs met the requirements.

### β-glucuronidase Staining

Plant tissues were incubated in β-glucuronidase (GUS) staining solution [10 μl X-Gluc stock (50 mg X-Gluc in 1 ml DMF) (1270MG100; BioFroxx, Germany), add 990-μl base solution (98.9 ml 100 mM PBS (pH 7), 0.164625 g K_3_[Fe(CN)_6_], 0.211195 g K_4_[Fe(CN)_6_]⋅3H_2_O, 100 μl Triton X-100, 0.37224 g Na_2_EDTA⋅2H_2_O)] for 6–8 h (for DR5:GUS) or overnight (for PIN:GUS) at 37°C. The samples were cleared using 95, 70, 50, and 25% ethanol sequentially and finally rinsed with distilled water. All the samples were observed using a fluorescence microscope (BX41, Olympus, Japan).

### 1-Naphthylacetic Acid and N-1-naphthylphthalamic Acid Treatment

The seeds were sown on 1/2 MS medium with 50 nM 1-Naphthylacetic acid (NAA) (HY-18570; MedChemExpress, United States) or 3 μM N-1-naphthylphthalamic acid (NPA) (N131601; Aladdin, United States) and grew vertically for seven days. Root length in all the experiments was measured using ImageJ.

### NaCl, Mannitol, and Abscisic Acid Treatment

The seeds were sown in a 1/2 MS medium with 125 mM NaCl (YongDa Chemical, Tianjin, China) and 200 mM mannitol (Sinopharm Chemical Reagent Co., Ltd., Shanghai, China) or 1 μM ABA (Yuanye Bio-Technology, Shanghai, China), and grew vertically for 7 days.

### Gene Ontology Enrichment Analysis

For function enrichment analysis, Gene Ontology (GO) analysis was conducted on the identified differently expressed genes (DEGs) (Ashburner et al., 2000) using online OmicShare tools.^1^ First, all the DEGs were mapped to GO terms in the Gene Ontology database,^2^ gene numbers were calculated for every term, and significantly enriched GO terms in the DEGs compared to genome background were defined by hypergeometric test. Calculated *p*-values underwent FDR correction with FDR ≤ 0.05 as the threshold. Finally, we filter out excessive terms in the three main categories [biological process (BP), MC, and cellular component (CC)].

### Accession Numbers

GenBank/EMBL accession numbers and *Arabidopsis* Genome Initiative locus identifiers for the genes mentioned in this article are as follows: *MAG2*, *At3g47700*.*1*; *MAL*, *At1g08400*; *AtSYP81*, *At1g51740*; *AtSec20*, *At3g24315*; *PIN4*, *At2g01420*; *PIN5*, *At5g16530*; *PIN7*, *At1g23080*; *AUX1*, *At2g38120*; *IAA1*, *At4g14560*; and *IAA3*, *At1g04240*.

## Results

### MAIGO2 and MAG2-Like Play an Important Regulatory Role in Plant Growth and Development With Functional Redundancy

**FIGURE 1:**
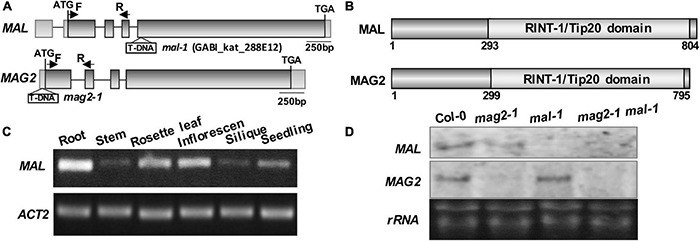
Structural diagram of *MAL* and T-DNA insertion mutant. **(A)** *MAG2* and *MAL* gene structures and T-DNA insertion sites in the mutants. The arrows indicate the position of real-time (RT)-PCR primers. **(B)** MAL and MAG2 protein structures and conserved domains. **(C)** RT-PCR determination of the tissue expression pattern of *MAL*. **(D)** Northern blot detection of the expression levels of *MAL* and *MAG2*.

Our previous study demonstrated that MAG2 plays a crucial role in ER export (Li et al., 2006, 2013). MAG2 has a homologous protein, MAG2-like (MAL) (Zhao et al., 2013; Zhao and Lu, 2014). Both of them have similar gene structure (Figure 1A) and protein structure that contain a conserved RINT-1/TIP20 domain (Figure 1B). Tissue expression determination revealed that *MAL* was expressed in all tissues, with highest level in roots, followed by rosette leaves, inflorescences, and seedlings, and with lowest level in stems and siliques (Figure 1C).

In order to analyze the function of *MAL*, we isolated a T-DNA insertion mutant, *mal-1*, in which T-DNA was inserted in the fourth exon (Figure 1A). Northern blot and RT-PCR analysis indicated that *MAL* expression was depleted in *mal-1* and reduced in *mag2-1*, but that *MAG2* expression had no significant change in *mal-1* (Figure 1D and Supplementary Figure 1A). In order to investigate the regulatory function MAG2 and MAL in plant development, we crossed *mal-1* with *mag2-1* to generate a double mutant. We also generated *TAP*-*MAL* overexpression (*MAL*/OE) plants. RT-PCR and immunoblot analysis indicated higher expression levels of *MAL* in *MAL*/OE lines (Supplementary Figures 1A,B).

**FIGURE 2:**
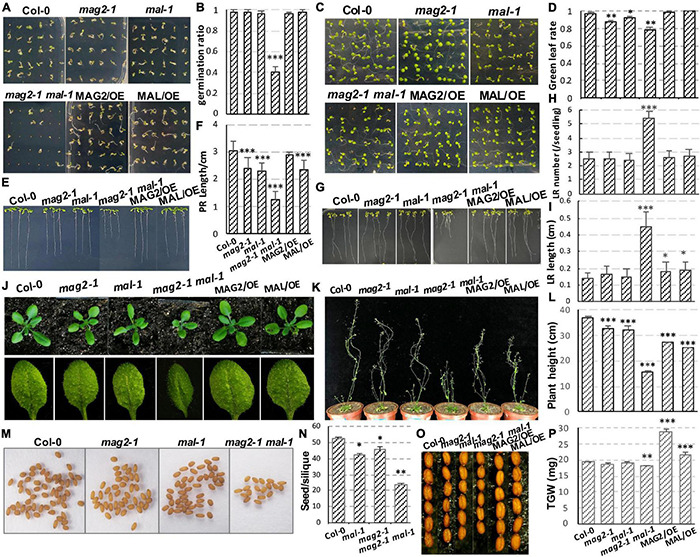
MAG2 and MAL regulate plant growth and development with redundancy. **(A)** Three-day-old seedlings grown in the 1/2 Murashige and Skoog (MS) medium. **(B)** Statistics of germination ratio in panel **(A)**. Values are means ± SD; *n* = 36, three repeats per sample. **(C)** Six-day-old seedlings grown in the 1/2 MS medium. **(D)** Statistics of green leaf ratio in panel **(C)**. Values are means ± SD; *n* = 36, three repeats per sample. **(E)** Seven-day-old seedlings grown in the 1/2 MS medium vertically. **(F)** Statistics of primary root length in panel **(E)**. Values are means ± SD; *n* = 30, three repeats per sample. **(G)** Fourteen-day-old seedlings grown in the 1/2 MS medium vertically. **(H)** Statistics of lateral root number of seedlings in panel **(G)**. Values are means ± SD; *n* = 10 PR, three repeats per sample. **(I)** Statistics of lateral root length in panel **(G)**. Values are means ± SD; *n* = 30, three repeats per sample. **(J)** Thirty-six-day-old plants and their rosette leaves. **(K)** Seventy-day-old plants. **(L)** Statistics of plant height in panel **(K)**. **(M)** Seeds in one silique from indicated lines. **(N)** Statistics of seed number per silique of panel **(M)**. **(O)** Seed size comparison. **(P)** Statistics of thousand grain weight. PR, primary root; LR, lateral root; TGW, thousand grain weight. *p < 0.05, ^**^p < 0.01, and ^***^p < 0.001. Significance was evaluated by Student’s *t*-test using the IBM SPSS Statistics 26 software. The seeds used in this study were all newly harvested.

The germination ratio of *mal-1* and *mag2-1* single mutants did not have significant change compared with that of the wild type (Figures 2A,B), but green leaf ratio was lower than that of the wild type (Figures 2C,D). The germination ratio and green leaf ratio of the *mal-1 mag2-1* double mutant were significantly reduced, but those of *MAG2*/OE and *MAL*/OE did not change significantly compared with those of the wild type (Figures 2A–D). The primary root length of seven-day-old seedlings of *mag2-1*, *mal-1*, *mal-1 mag2-1* mutants and *MAL*/OE line was significantly shorter than that of wild type, especially *mal-1 mag2-1* double mutant. However, there was no significant difference between wild type and *MAG2*/OE (Figures 2E,F). Noticeably, the lateral root (LR) number of 14-day-old seedlings of the *mal-1 mag2-1*double mutant was higher than that of the wild type, but there was no significant difference among the other lines (Figures 2G,H). The LR length of *mal-1 mag2-1* and MAG2/OE and MAL/OE lines were significantly longer than that of wild type, especially the double mutant was more than twice of wild type. However, there was no significant difference between the *mal-1* and *mag2-1* single mutants and the wild type (Figure 2I). The aerial part and rosette leaves of 36-day-old plants of *mag2-1*, *mal-1*, and *mal-1 mag2-1* were smaller than those of the wild type (Figure 2J), especially the double mutant, while the 70-day-old plant height of all the mutants and OE lines were shorter than that of the wild type, especially the double mutant (Figures 2K,L).

The seed number per silique of *mag2-1*, *mal-1*, and *mal-1 mag2-1* was significantly less than that of the wild type, especially *mal-1 mag2-1* (Figures 2M,N). The seed size of *mag2-1*, *mal-1*, and *mal-1 mag2-1* was smaller than that of the wild type, but that of *MAG2*/OE and *MAL*/OE was larger than that of the wild type (Figure 2O). Consistent with this, the thousand grain weight (TGW) of *mag2-1* and *mal-1 mag2-1* was significantly lower than that of the wild type, that of *MAG2*/OE and *MAL*/OE was significantly higher than that of the wild type, and that of *mal-1* was similar to that of the wild type (Figure 2P). The above results suggest that both MAG2 and MAL are involved in regulation of plant growth and development. The fact that the phenotypes of the *mal-1 mag2-1* double mutant were more serious than those of the *mal-1* and *mag2-1* single mutants suggest that both MAG2 and MAL play important roles in plant growth and development with functional redundancy.

### MAIGO2 and MAG2-Like Regulate Protein Transport With Redundancy

**FIGURE 3:**
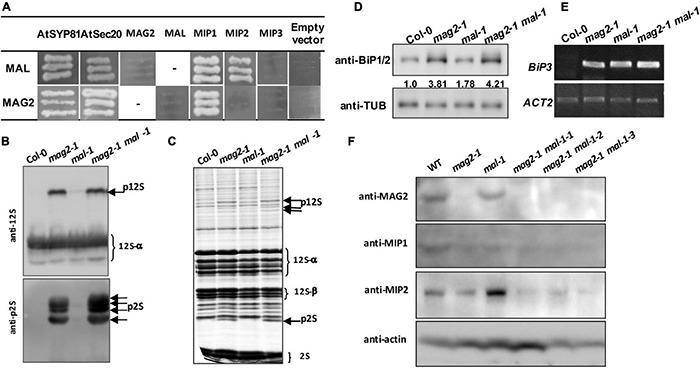
MAG2 and MAL regulate protein export from the endoplasmic reticulum (ER). **(A)** Yeast two hybrid detection of MAL interactors. Detection of combinations of both *MAL/pGADT7* and *MAL/pGBKT7* with corresponding constructs was performed. AtSYP81 vs. AtSec20 served as positive control, whereas MAL/MAG2 vs. empty vectors served as negative control. −, Not performed. **(B)** Immunoblot detection of precursors of seed storage proteins. **(C)** Profile of whole seed proteins. **(D)** Immunoblot detection of BiP1/BiP2 proteins. Statistics of relative band concentration (presents protein abundance) is indicated in number below the bands (BiP/TUB, measured with ImageJ). **(E)** RT-PCR determination of *BiP3* expression. **(F)** Immunoblot detection of MAG2, MIP1, and MIP2 in 7-day-old seedlings of indicated lines. 12S, 12S globulins; 2S, 2S albumins; p12S, precursors of 12S globulins; p2S, precursors of 2S albumins.

Our previous research clarified that MAG2 forms a complex with MIP1, MIP2, and MIP3 to regulate Golgi-to-ER retrograde transport (Li et al., 2006, 2013). MAG2 also interacts with ER-localized Qa-SNARE AtSYP81 and Qb-SNARE AtSec20 to promote membrane fusion (Li et al., 2013). In the *mag2*, *mip1*, *mip2*, and *mip3* mutants, ER export of SSP precursors is blocked, which results in accumulation of proteins inside the ER and serious ER stress (Li et al., 2006, 2013; Zhao et al., 2018; Guan et al., 2021). In order to explore the role of MAL in vesicle transport, we first performed yeast two hybrid analysis to detect the interaction between MAL and SNARE and MAG2 complex subunits. The results indicated that MAL interacted with AtSYP81, AtSec20, MIP1, and MIP2, and that MAG2 interacted with AtSYP81, AtSec20, and MIP1 (Figure 3A), suggesting that MAL has the ability to form a complex with MIP subunits to regulate arriving vesicle tether to the ER membrane, maybe coordinately with SNAREs.

Immunoblot analysis revealed that a trace amount of SSP precursors accumulated in the *mal-1* seeds, and that numerous precursors accumulated in the double mutant seeds and were more than those in *mag2-1* (Figures 3B,C). This suggests that MAL plays a minor role in protein transport and that it is functionally redundant with MAG2. Since abnormal accumulation of proteins in the ER lumen induces ER stress (Li et al., 2006, 2013; Zhao et al., 2018; Guan et al., 2021), we detected the expression of ER stress markers. Western blot of BiP1/2, a common ER stress marker, indicated that their protein accumulation was significantly increased in all the mutants, especially in the *mal-1 mag2-1* double mutant (Figure 3D). Moreover, RT-PCR determination of *BiP3*, and ER stress-specific marker, indicated that the transcription of *BiP3* also increased significantly in all the mutants (Figure 3E). These results suggest that protein export from the ER is blocked, inducing ER stress in *mal-1*. To further clarify the function of MAL, we detected the protein accumulation of MAG2 complex subunits in the *mal-1* mutant. As shown in Figure 3F, in *mal-1*, the protein levels of MAG2 and MIP1 decreased, while that of MIP2 increased; in *mag2-1*, the protein levels of both MIP1 and MIP2 decreased; in the *mal-1 mag2-1* double mutant, MIP1 and MIP2 decreased more than in the single mutants, indicating that both MAG2 and MAL affect the stability of the MAG2 complex. The above results suggest that MAL also plays a role in protein transport, that MAG2 function is dominant, and that they are functionally redundant.

### Proteomics Analysis of Microsomal Membrane Proteins in the *mag2-1 mal-1* Double Mutant

**FIGURE 4:**
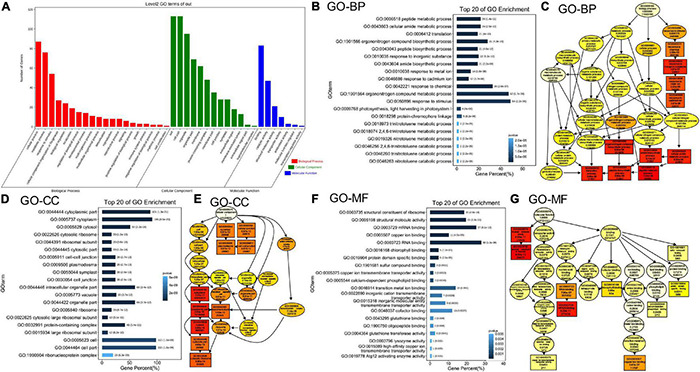
Gene Ontology (GO) classification of the differently accumulated proteins (DAPs). **(A)** DAP distribution in three GO terms of biological process (BP), cellular component (CC), and molecular function (MF). X axis represents GO terms. Y axis represents number of genes. **(B)** Top 20 GO enrichment terms in BP. X axis represents gene percentage. Y axis represents GO terms. The number on each column indicates DAP number, followed by *p*-value in the brackets. The same in panel **(D,F)**. **(C)** The functional categories of GO terms in BP are shown as a diagram. **(D)** Top 20 GO enrichment terms in CC. **(E)** The functional categories of GO terms in CC. **(F)** Top 20 GO enrichment terms in MF. **(G)** The functional categories of GO terms in MF.

To further elucidate the effects of simultaneous depletion of MAG2 and MAL on cellular activities, we performed a proteomics analysis using extracted microsomal fraction from roots of 7-day-old seedlings by label-free identification. A total of 4,546 proteins from both the wild type and *mag2-1 mal-1* were identified, 515 of which were DAPs in *mag2-1 mal-1* compared with those in the wild type. The DAPs were filtered by a change ratio >1.2 or *p* ≤ 0.05, and 124 DAPs met the requirements (Supplementary Table 1). Then, the set of 124 DAPs was subjected to GO analysis to achieve a broader functional characterization. As a result, the DAPs were classified into 40 subcategories within three main categories: 20 subcategories in BP, 13 in CC, and 7 in molecule function (MF) (Figure 4A). In total, 110 DAPs were associated with BP terms (GO:0008150), 115 DAPs were associated with CC terms (GO:0005575), and 107 DAPs were associated with MF terms (GO:0003674) (Supplementary Table 2). Among these, one DAP could be assigned to more than one category. In BP, the most enriched pathways were response to stimulus (GO:0050896) (54 DAPs), organonitrogen compound metabolic process (GO:1901564) (52 DAPs), response to chemical (GO:0042221) (36 DAPs), organonitrogen compound biosynthetic process (GO:1901566) (31 DAPs), peptide metabolic process (GO:0006518) (24 DAPs), and amino metabolic process (GO:0043603) (24 DAPs) (Figure 4B). In CC, the most enriched pathways were cell (GO:0005623) (113 DAPs), cell part (GO:0044464) (113 DAPs), cytoplasm (GO:0005737) (105 DAPs), cytoplasm part (GO:0044444) (101 DAPs), intracellular organelle part (GO:0044446) (69 DAPs), and organelle part (GO:0044422) (69 DAPs) (Figure 4D). In MF, the most enriched pathways were RNA binding (GO:0003723) (30 DAPs), mRNA binding (GO:0003729) (27 DAPs), structural molecule activity (GO:0005198) (21 DAPs), structural constituent of ribosome (GO:0003735) (20 DAPs), transition metal ion binding (GO:0046914) (15 DAPs), and cofactor binding (GO:0048037) (13 DAPs) (Figure 4F). The functional categories of GO terms of BP, CC, and MF were shown as a diagram (Figures 4C,E,G). These results indicate that metabolism, biosynthesis, signaling, and environmental response were affected in the *mag2-1 mal-1* double mutant.

### Depletion of MAIGO2 and MAG2-Like Affects Intracellular Transport

**FIGURE 5:**
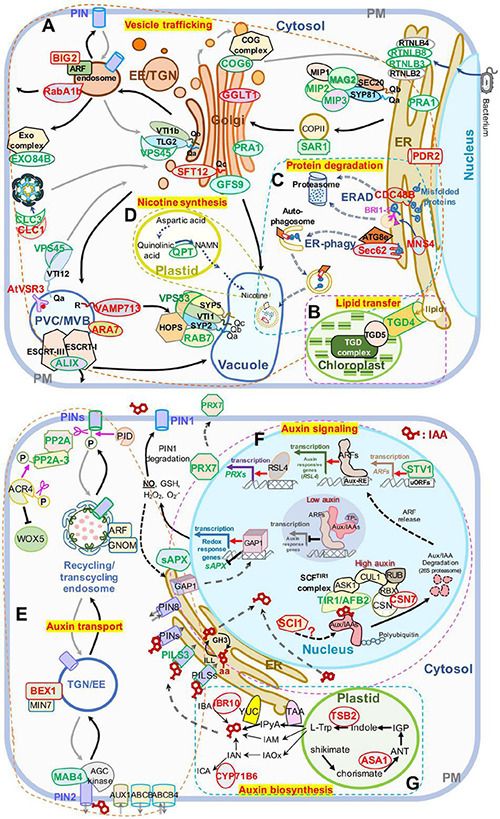
Schematic presentation of DAPs enriched in panel **(A–D)** vesicle trafficking and **(E–G)** auxin-related pathways. **(A)** Intracellular vesicle trafficking pathways. **(B)** Lipid transfer pathways. **(C)** Protein degradation pathways including ERAD and ER-phagy. **(D)** Nicotine biosynthesis pathways. **(E)** Auxin transport pathways. **(F)** Auxin signaling pathways. **(G)** Auxin biosynthesis pathways. Protein names in red and green represent increased and decreased DAP abundances, respectively. The ranges of panel **(A–G)** are defined by frames with dashed lines. AUX1, auxin resistant 1; ABCA, ATP binding cassette; ANT, anthranilate; AUX/IAAs, auxin/indole-3-acetic acid proteins; ARFs, auxin response factors; Aux-RE, auxin response element; ASK1, *Arabidopsis* serine/threonine kinase 1; ACR4, *Arabidopsis* crinkly 4; ATG8e, autophagy 8e; ALIX, ALG-2 interacting protein-X; BEX1/MIN7, bfa-visualized exocytic trafficking defective1/hopm interactor 7; CSN, COP9 signalosome subunit 7; CUL1, cullin 1; CYP71B6, cytochrome p450 71 B6; CDC48B, cell division cycle 48 B; COG6, conserved oligomric Golgi complex 6; CLC1, clathrin light chain 1; CLC3, clathrin light chain 3; EXO84B, exocyst complex component 84 B; EE, early endosomes; ER, endoplasmic reticulum; ERAD, endoplasmic reticulum (ER)-associated degradation; GAP1, GTPase-activating protein 1; GFS9, green fluorescent seed 9; GGLT1, golgi GDP-L-galactose transporter 1; IGP, indole-3-glycerol phosphate; IAOx, indole-3-acetaldoxime; IAM, indole-3-acetamide; IPyA, indole-3-pyruvic acid; ICA, indole-3-carboxaldehyde; IAN, indole-3-acetonitrile; IBA, indole-3-butyric acid; IBR10, indol-3-butyric acid response 10; L-Trp, L-tryptophan; MAB4, macchi-bou 4; MNS4, mannosidase 4; PIN, PIN-FORMED; PRX7, peroxidase 7; PILS3, PIN-LIKES 3; PP2A, protein phosphatase 2A; PID, PINOID; PRA1, prenylated rab acceptor 1; PDR2, phosphate deficiency response 2; QPT, quinolinate phoshorbosyl transferase; RBX, ring-box 1; RUB, ubiquitin-related protein; RSL4, root hair defective 6-like 4; RTNLB3, reticulon-like protein B3; reticulon-like protein B8; RABA1B, rab GTPase homolog A1B; RTNL3, reticulon 3; sAPX, stromal ascorbate peroxidase; SCI, stigma/style cell-cycle inhibitor 1; TGN, trans-Golgi network; TIR1/AFB2, transport inhibitor response 1/auxin signaling F-box 2; TPL, topless; TSB2, tryptophan synthase beta-subunit 2; TAA, tryptophan aminotransferase of *Arabidopsis*; TGD4, trigalactosyldiacylglycerol 4; uORF, upstream open reading frame; WEI2, weak ethylene insensitive 2; VSR3, vacuolar sorting receptor 3; VPS45, vacuolar protein sorting 45; VPS33, vacuolar protein sorting 33; YUC, YUCCA flavin-containing monooxygenases; YUC4, YUCCA4. A “P” in a circle indicates phosphorylation; pink scissors represent dephosphorylation; the pink arrow represents phosphorylation; the red arrows represent transcriptional activation; the black T-shape indicates transcriptional inhibition; the white blocks with lattices represent regulatory cis-elements; the red structural formula represents IAA molecule, and the one with –aa represents IAA–amino acid conjugates; right-angled arrows represent transcription products; the long gray and black arrows represent cycling transport pathways.

We first extracted DAPs related to vesicle trafficking (Supplementary Table 3) and restored their functional location (Figure 5A). In ER-Golgi transport, the protein levels of SAR1 (initiates coat assembly in COPII vesicles) (Saito et al., 2017) in the anterograde pathway, and MIP2 and MIP3 (MAG2 complex subunits) (Li et al., 2006, 2013), and RTNLB3 and RTNLB8 (RTN) (RTN complex subunits) (Huang et al., 2018), in the retrograde pathway were decreased. In intra-Golgi trafficking, the protein level of conserved oligomric golgi complex 6 (COG6) (COG complex subunit) (Ungar et al., 2002; Zolov and Lupashin, 2005; Trahey and Hay, 2010) was decreased, while the protein levels of Golgi-localized galactose transporter GGLT1 (Sechet et al., 2018) and phosphate deficiency response 2 (PDR2) (mediates manganese transport into the ER) (Alvim Kamei et al., 2008) were increased. On the secretory and endocytic/recycling pathway, the protein levels of PRA1 (with multiple localization of ER, Golgi, and endosome, functioning in both secretory and endocytic pathways) (Alvim Kamei et al., 2008), the trans-Golgi network (TGN)-localized SM protein AtVPS45 (binds with Qa-AtTLG2 and Qb-AtVTI1b to mediate endosome-to-TGN transport) (Bassham et al., 2000), the PM-localized EXO84B (a subunit of exocyst complex that tethers Golgi/TGN-derived vesicles to the PM) (Heider and Munson, 2012; Saeed et al., 2019), and a clathrin light chain protein, CLC3, weredecreased; while the protein levels of CLC1, another CLC protein, RabA1b/BEX5, a TGN/EE-localized small GTPase (regulates TGN-to-PM trafficking) (Wang et al., 2013; Majeed et al., 2014), and BIG2/BEN3, an guanine-nucleotide exchange factor of ADP-ribosylation factor (ARF GEF) protein (regulates PIN1 secretion) (Kitakura et al., 2017), were increased. In the vacuole-targeting pathway, the protein levels of the Golgi-localized Qc-SNARE AtSTF12 (regulates Na^+^ sequestration in vacuoles under salt and osmotic stress) (Tarte et al., 2015), PVC/MVB-localized ARA7 (a Rab5 homolog) (Lee et al., 2004), and R-SNARE VAMP713, which interacts with the vacuolar-tether complex HOPS to regulate vacuole targeting (Takemoto et al., 2018), and the vacuolar sorting receptor VSR3 (functions in vacuolar cargo sorting) (Lee et al., 2013; Ichino et al., 2014) were increased; while the Golgi-localized GFS9 (involved in proteins and phytochemical transport to vacuoles) (Ichino et al., 2014), MVB/PVC-localized ALIX, the bridge protein of ESCRT-I and ESCRT-III complexes (essential for vacuolar targeting) (Shen et al., 2016), RAB7 and a HOPS subunit, VPS33 (also a SM protein) (both proteins bind vacuolar SNARE complexes to facilitate membrane fusion) (Lobingier and Merz, 2012), were decreased. The protein level of trigalactosyldiacylglycerol 4 (TGD4), which is localized in ER-chloroplast membrane contact sites and mediates the transfer of lipid precursors from the ER to chloroplast for biogenesis of photosynthetic membranes (reviewed by Fan et al., 2015), was also decreased (Figure 5B). All the influences on diverse pathways suggest that blocking of protein export from the ER in *mag2-1 mal-1* affects subsequent vesicle trafficking processes.

### MAIGO2 and MAG2-Like Deficiency Influenced Protein Quality Control

The ER is crucial for maintenance of cellular homeostasis, because its functions in various cellular processes, such as folding and initial modification of secretory and transmembrane proteins. Misfolded and unfolded proteins that accumulate in the ER lumen induce ER stress. To maintain ER homeostasis, several strategies have been evolved, including unfolded protein response (UPR), ER-associated degradation (ERAD), and ER-phagy (Chen et al., 2020). As shown in Figure 5C, the protein levels of ER-localized CDC48B ATPase (extracts unfolded/misfolded proteins from the ER lumen and membrane for targeting to proteasomes) (Wu and Rapoport, 2018) and MNS4 mannosidase (promotes BRI1-5 ubiquitylation and degradation) (Hüttner et al., 2014) in the ERAD pathway, and Sec62, an ER-phagy receptor (coordinates with ATG8e to engulf misfolded proteins into autophagosomes for vacuolar degradation) (Hu et al., 2020) were increased. These changes have probably resulted from blocking of protein export from the ER, suggesting that MAG2 and MAL are important for protein quality control. Interestingly, the quinolinic acid phosphoribosyl transferase (QPT), which is essential for pyridine nucleotide cycle and biosynthesis of alkaloid nicotine (Eads et al., 1997; Sinclair et al., 2000) decreased in *mag2-1 mal-1* (Figure 5D).

### MAIGO2 and MAG2-Like Affect Abundance of Regulators Related to Auxin Biosynthesis, Transport, and Signaling

Since the growth and development of the *mag2-1 mal-1* double mutant were seriously affected, we then focused on auxin-related DAPs and plant phenotypic analysis. We first extracted auxin-related DAPs (Supplementary Table 4) and restored their function (Figures 5E–G).

In auxin transport pathways (Figure 5E), the protein levels of the phosphatase PP2A (works antagonistically with PINOID kinase in PIN cycling) (Feraru and Friml, 2008; Grones et al., 2018), PP2A-3, a catalytic subunit of PP2A holoenzymes (dephosphorylates ACR4, a PM-localized receptor kinase controlling WOX5 expression) (Kong et al., 2015; Yue et al., 2016), PILS3, an ER-localized auxin transporter, and MAB4, an interactor of PIN1 and PIN2 (coordinates with AGC kinases to regulate PIN polar localization) (Glanc et al., 2021) were decreased, while the protein level of the TGN/EE-localized BEX1, an ARF protein (facilitates PIN recycling to the PM) (Tanaka et al., 2014), was increased. These results suggest that MAG2 and MAL might affect auxin transport by influencing polar localization maintenance of auxin carriers.

In auxin signaling pathways (Figure 5F), the protein levels of TIR1/AFB2, a subunit of the SCF^*TIR*1^ complex (triggers proteasomal degradation of Aux/IAA to release ARFs for transcriptional activation of auxin-responsive genes such as *RSL4*) (Pires et al., 2013; Mangano et al., 2017), *PRX7*, a class III peroxidase activated by RSL4 (Vijayakumar et al., 2016; Marzol et al., 2017), STV1/RPL24, which regulates the expression of auxin responsive genes (Sessions et al., 1997; Hardtke and Berleth, 1998), and sAPX, the stromal APX regulated by GAP1/ANAC089 (Klein et al., 2012; Yang et al., 2014) which triggers production of nitric oxide (NO) to regulate auxin transport in a PIN1-dependent manner (Fernández-Marcos et al., 2011), were decreased, while the protein levels of CSN7, a subunit of the CSN complex regulating AUX/IAA degradation (Serino and Deng, 2003; Mergner and Schwechheimer, 2014), and SCI1, which affects the transcription of auxin-responsive genes such as *IAAs* (Serino and Deng, 2003; Mergner and Schwechheimer, 2014), were increased. In auxin biosynthesis pathways (Figure 5G), IAA is synthesized mainly from L-Trp precursors, which are generated *via* the shikimate pathway. ASA1, an anthranilate synthase subunit that catalyzes shikimate to produce anthranilate (ANT) (Radwanski and Last, 1995; Li et al., 2020), TSB2, a tryptophan synthase subunit that catalyzes the formation of Trp from indole (Wang et al., 2015; Li et al., 2020), CYP71B6, a monooxygenase that converts indole-3-acetonitrile (IAN) to ICA (Böttcher et al., 2014; Müller et al., 2019; Pastorczyk et al., 2020), and IBR10, which can convert IBA to IAA (reviewed by Strader and Bartel, 2011), were increased in *mag2-1 mal-1*. These results suggest that MAG2 and MAL might affect auxin signaling and biosynthesis by influencing the abundance of regulators.

### Auxin Distribution Was Affected in Different Manner in *mag2-1* and *mal-1*

**FIGURE 6:**
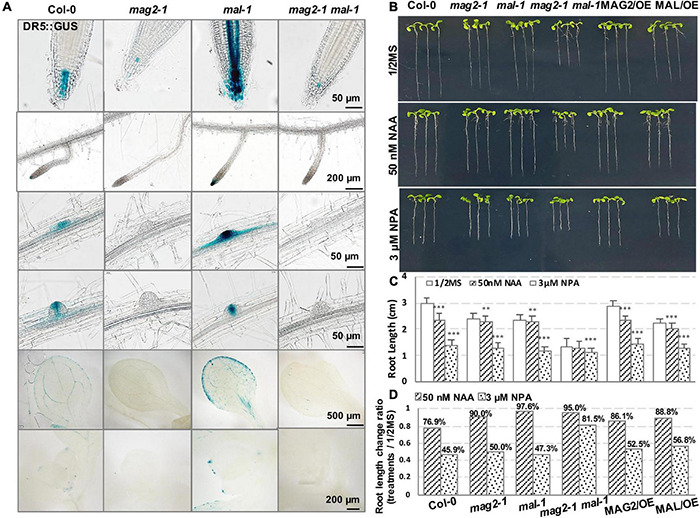
MAG2 and MAL regulate auxin distribution. **(A)** Tissue expression pattern of DR5:GUS. Bars are as shown. **(B)** Seven-day-old seedlings grown in the 1/2 MS medium with 50 nm NAA or 3 μM NPA. **(C)** Statistics of root length in panel **(B)**. Values are means ± SD; *n* = 30, three repeats per sample. **(D)** Statistics of root length ratio before and after treatment in panel **(C)**. ^**^p < 0.01, and ^***^P < 0.001. Significance was evaluated by Student’s *t*-test using IBM SPSS Statistics 26.

Then, we determined auxin distribution in the mutants using an auxin response marker, DR5:GUS, which was introduced into each mutant by crossing. Chemical staining indicated that DR5:GUS signal was distributed in quiescent cells (QCs) and columella cells in primary and lateral root tips, lateral root primordium, cotyledon veins and margin, and true leaf tips in the wild type. However, in *mag2-1*, the DR5:GUS signal was significantly reduced, only observed in a few columella cells in primary root tips. Unexpectedly, the expression pattern of DR5:GUS in *mal-1* was completely different. In *mal-1* primary roots, the DR5:GUS signal increased significantly not only in QCs and columella cells but also in stele cells. Moreover, increased GUS signals were also observed in lateral root tips, lateral root primordium, cotyledon veins and margin, and true leaf tips, while in the *mag2-1 mal-1* double mutant, DR5:GUS distribution was similar to that in *mag2-1* (Figure 6A). These results suggest that knockout of MAG2 and MAL affects auxin level and distribution, but that the two homolog proteins might play different regulatory roles in auxin distribution.

We further detected the auxin response of the mutants and OE lines. Application of 50 nM of NAA, a synthetic auxin analog, inhibited the growth of primary roots of 7-day-old seedlings. In the wild type, root length decreased by more than 20%, while the reduction in the root length of mutants and OE lines was much less than that in the wild type (Figures 6B–D). These results suggest that MAG2 and MAL are involved in auxin response.

Then, we checked polar auxin transport (PAT). Application of 3 μM of NPA, an auxin transport inhibitor, inhibited the growth of primary roots of 7-day-old seedlings. Reduction in the root length of the *mag2-1* and *mal-1* single mutants and the OE lines was less than that of the wild type, while the root growth of the *mag2-1 mal-1* double mutant was not sensitive to the inhibition of 3 μM NPA treatment (Figures 6B–D). These results suggest that PAT was affected in the mutants and OE lines, especially in the *mag2-1 mal-1* double mutant.

### PIN-FORMED Abundance Was Affected in the Mutants

**FIGURE 7:**
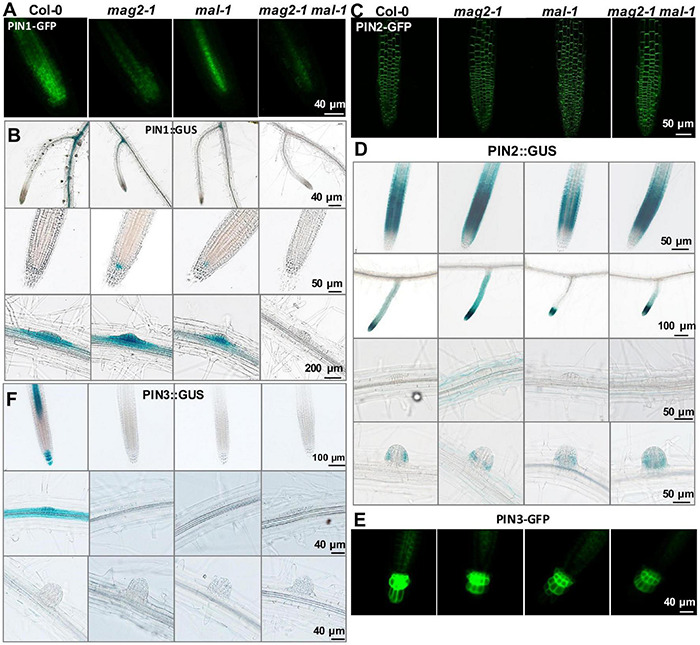
MAG2 and MAL regulate the expression of auxin transporters. Confocal images of **(A)** PIN1-GFP, **(C)** PIN2-GFP, and **(E)** PIN3-GFP in primary roots. **(B,D,F)** Expression pattern of **(B)** PIN1:GUS, **(D)** PIN2:GUS, and **(F)** PIN3:GUS in 7-day-old seedlings grown in 1/2MS medium tissues. Bars are as shown.

To clarify the mechanisms underlying MAG2 and MAL regulation in auxin transport, we introduced the cassettes of PIN1:GUS, PIN1-GFP, PIN2:GUS, PIN2-GFP, PIN3:GUS, and PIN3-GFP into the mutants by crossing, and we observed their distribution. PIN1 is localized on cell basal side in root stele and stem vascular tissue, as well as lateral root primordium (LRP) (Omelyanchuk et al., 2016). Compared with the wild type, PIN1-GFP signals decreased significantly in stele cells in *mag2-1*, *mal-1*, and *mag2-1 mal-1*, especially in the *mag2-1 mal-1* double mutant (Figure 7A). PIN1:GUS in stele cells of primary roots decreased significantly in *mag2-1*, *mal-1*, and *mag2-1 mal-1*, especially in *mag2-1 mal-1*. Interestingly, PIN1:GUS expression increased in QC cells in primary roots of *mag2-1* and *mal-1*, especially *mag2-1*. No signal was observed in the *mag2-1 mal-1* double mutant (Figure 7B). In wild-type LRP, PIN1:GUS evenly distributed in all cells, but in *mag2-1* LRP, PIN1:GUS signals increased in the basal layer. Conversely, in *mal-1* LRP, PIN1:GUS signals decreased in the outer layer. No signals were detected in the *mag2-1 mal-1* double mutant (Figure 7B). PIN2 is mainly expressed in cortical and epidermal cells in root tips and is involved lateral root development (Chen et al., 1998; Zhou and Luo, 2018). In *mag2-1* and *mal-1*, PIN2-GFP localization and abundance did not change significantly, but in the *mag2-1 mal-1* double mutant, PIN2-GFP abundance likely increased (Figure 7C). PIN2:GUS signals increased in cortical and epidermal cells in primary and lateral root tips of *mag2-1* and *mag2-1 mal-1* but decreased in *mal-1* (Figure 7D). In the early LR development stage, PIN2:GUS in *mag2-1* tended to accumulate in basal layers compared with that in the wild type, but in *mal-1*, PIN2:GUS signals became weaker, whereas in the *mag2-1 mal-1* double mutant, GUS signals became higher and diffused (Figure 7D). PIN3 is distributed in root columella and stele cells (Li et al., 2015), participating in primary root development and lateral root formation in early steps (Zhou and Luo, 2018). In *mag2-1*, *mal-1*, and *mag2-1 mal-1*, PIN3-GFP abundance in columella and stele cells reduced, especially in the *mag2-1 mal-1* double mutant (Figure 7E). PIN3:GUS was expressed in stele, columella, and LRP cells in the wild type, but almost no signal was detected in all the mutants (Figure 7F). We further determined the expression of *AUX1*, part of *PIN* and *IAA* genes. The results indicate that the expression of *IAA1* increased and that of *IAA3* reduced slightly (Supplementary Figure 1C). The alteration in abundance of PIN1, PIN2, and PIN3, and expression of *IAAs* in the mutants might lead to abnormal auxin transport and distribution and affect lateral root development. Combined with the proteomics results, it is suggested that auxin transport and signaling are disturbed in MAG2- and MAL-deficient mutants.

### MAIGO2 and MAG2-Like Are Involved in Plant Stress Response

It was observed that in the early stage of germination, the seedlings of *mag2-1*, *mal-1*, *mag2-1 mal-1*, *MAG2*/OE, and *MAL*/OE lines accumulated higher levels of anthocyanins than those of the wild type (Supplementary Figure 1D). Anthocyanins are antioxidants that protect plants from growth inhibition and cell death by scavenging abiotic stress-induced ROS, thereby enabling plant adaption to abiotic stress (Naing and Kim, 2021). The higher accumulation of anthocyanins suggested loss of ROS homeostasis in the mutants and OE plants.

**FIGURE 8:**
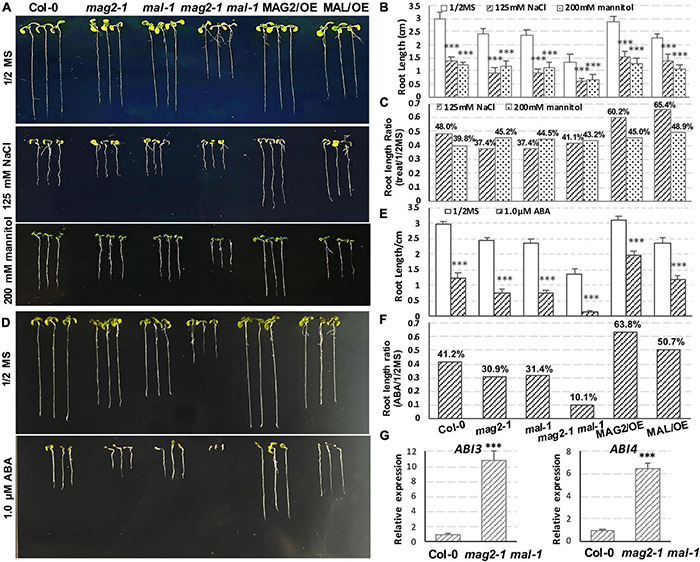
MAG2 and MAL regulate plant stress response. **(A)** Seven-day-old seedlings grown in the 1/2 MS medium with 125 mM NaCl or 200 mM mannitol. **(B)** Statistics of root length in panel **(A)**. Values are means ± SD; *n* = 30, three repeats per sample. **(C)** Statistics of root length ratio before and after treatment in panel **(C)**. **(D)** Seven-day-old seedlings grown in the 1/2 MS medium with 1 μM ABA. **(E)** Statistics of root length in panel **(D)**. Values are means ± SD; *n* = 30, three repeats per sample. **(F)** Statistics of root length ratio before and after treatment in panel **(E)**. **(G)** RT-qPCR determination of relative expression level of*ABI3* and *ABI4*. Two independent experiments per sample, and three repeats per experiment. *p < 0.05, ^**^p < 0.01, and ^***^p < 0.001. Significance was evaluated by Student’s *t*-test using IBM SPSS Statistics 26.

In order to explore the function of MAG2 and MAL in plant response to environmental stress, we performed salt, osmotic, and ABA treatments. In the 125-mM NaCl treatment, reduction of root length of the mutants was more than that of the wild type, but that of the OE lines was less than that of the wild type (Figures 8A–C). In the 200-mM mannitol treatment, reduction of root length of the mutants and OE lines was less than that of the wild type (Figures 8A–C). In the 1-μM ABA treatment, reduction of root length of the mutants was higher, and that the OE lines was less than that of the wild type (Figures 8D–F). Since ABA signaling was disrupted in *mag2-1* (Zhao et al., 2018), we checked the expression levels of *ABI3* and *ABI4* in *mag2-1 mal-1*. As shown in Figure 8G, the expression of *ABI3* and *ABI4* was significantly elevated in *mag2-1 mal-1*. All these results suggest that MAG2 and MAL play important roles in regulation of plant stress response.

## Discussion

### MAIGO2 and MAG2-Like Play Important Roles in Plant Development With Functional Redundancy and Division

Our previous study clarified that MAG2 forms a tethering complex with MIP1, MIP2, and MIP3 to regulate protein export from the ER. Deficiency of any subunit of the complex leads to the formation of a novel cell structure (we call it “*mag* Body”), which contains precursors of SSPs and the ER, BiP, and PDI. *mag* Bodies are trapped inside the ER lumen and induce severe ER stress (Li et al., 2006, 2013; Zhao et al., 2018). In this study, we investigate the function of MAL and compared it with that of its homologs protein, MAG2. As expected, MAL also plays roles in vesicle trafficking, plant development, and environmental stress response, and it was functionally redundant with MAG2. MAL and MAG2 deficiency significantly affected the stability of the MAG2 complex (Figure 3F), indicating that MAL might form a complex with MIP proteins to regulate vesicle transport when MAG2 is deficient, or in different developmental stages or tissues.

One observation that attracted our attention was the different performance of MAL and MAG2 on auxin transport. The DR5:GUS signals were reduced significantly in *mag2-1* but, conversely, were elevated substantially in *mal-1*, and the *mag2-1 mal-1* double mutant displayed a trend similar to *mag2-1* (Figure 6). Similarly, the opposite phenotypes were also observed in PIN2:GUS distribution in roots. The PIN2:GUS signals were increased significantly in *mag2-1*, while they were decreased markedly in *mal-1* in root elongation zones. The *mag2-1 mal-1* double mutant displayed a trend similar to *mag2-1* (Figure 7D). Also, PIN1:GUS expression level in LRP in *mag2-1* was elevated but reduced in *mal-1* (Figure 7B). These phenotypes suggest that MAL and MAG2 have a functional division in regulating auxin transport, and that their functions might be opposite: MAG2 plays a positive role, while MAL plays a negative role, and MAG2 is dominant. However, the speculation needs more evidence to be confirmed.

The *mag2* and *mip* single mutants as well as their double mutants such as *mag2-1 mip3-1* and *mip2-1 mip3-1* have a distorted response to environmental stresses (Zhao et al., 2018). The single and double mutant seeds have reduced protein qualities, germination activities, and longevity, since they have reduced content of mature SSPs, which could protect cell components and cell structures from oxidative stress during deposition. The blocking of vesicle transport in the *mag2* and *mip* single and double mutants disturb endomembrane function and ABA signaling. The expression levels of *ABI3*, *ABI4*, and *ABI5* was altered significantly compared with that of the wild type under normal and stress conditions (Zhao et al., 2018). Consistent with these, the expression of *ABI3* and *ABI4* in *mag2-1 mal-1* was also altered significantly (Figure 8G), suggesting that serious blocking of ER export is bound to affect ABA signaling.

### MAIGO2 and MAG2-Like Regulate Auxin Homeostasis by Controlling Golgi-to-Endoplasmic Reticulum Vesicle Trafficking

Since ER export is blocked in *mag2-1*, the function of ER is seriously disrupted. Numerous newly synthesized proteins are trapped inside the ER lumen and form a novel cell structure, *mag* Body, and subsequently induce severe ER stress (Li et al., 2006, 2013; Zhao et al., 2018). The *mag2-1 mal-1* double mutant has more serious transport defects such as more SSP precursors and higher ER stress than the *mag2-1* single mutant, thus ER function disorder should be more serious. A large amount of DAPs in vesicle trafficking pathways represent the severity of the disorder (Figure 5A). The DAPs were distributed not only in the ER-Golgi COPI- and COPII-mediated pathways but also in the late secretion and recycling pathways as well as vacuole targeting pathways. This reflected the close correlation among the transport pathways. The ER is the initial point of secretory pathway and is important for ion homeostasis, quality control of newly synthesized proteins, lipid biosynthesis and transfer, and organelle communication (Borgese et al., 2006). The serious protein export jam and ER stress in *mag2-1 mal-1* double mutant definitely disrupted ER homeostasis and functions, and affected the abundance of regulators of vesicle trafficking (Figure 5A), ERAD and ER-phagy pathways (Figure 5B), and lipid transfer system (Figure 5C). As a consequence, cellular function and integrity as well as plant development were seriously affected.

Another spectacular change was the large amount of DAPs in auxin transport, signaling, and biosynthesis pathways (Figures 5E–G). Auxin homeostasis is coordinately regulated by multiple processes such as IAA biosynthesis, conjugation, transport, and signaling as. However, the controlling mechanisms of IAA homeostasis is elusive because of the complexity of combination of diverse pathways and spatiotemporal (different organs and developmental stages) and environmental factors. The two-step pathway converting tryptophan (Trp) to IAA is a highly conserved auxin biosynthetic pathway. TAA aminotransferases catalyze tryptophan to IPyA, and then YUC monooxygenases convert IPyA to IAA (Stepanova et al., 2008; Cao et al., 2019). Flower-specific YUC4.2 is the first reported ER membrane-anchored monooxygenase (Kriechbaumer et al., 2012). In *Arabidopsis* and maize, about half of TAA/TAR and YUC family enzymes such as TAR2, YUC3, YUC5, YUC7, YUC8, and YUC9, are localized in the ER membrane (Kriechbaumer et al., 2015, 2016). These enzymes are actively involved in auxin biosynthesis (Kriechbaumer et al., 2016; Poulet and Kriechbaumer, 2017). Moreover, about 20% of the total IAA biosynthetic activity was detected in a purified microsomal membrane fraction (Kriechbaumer et al., 2015, 2016). Thus, the ER could be considered as a platform for auxin biosynthesis. The abnormal protein accumulation inside the ER in the *mag2-1 mal-1* double mutant will definitely affect the function of these auxin biosynthesis-related proteins.

Endoplasmic reticulum-localized PIN5, PILS2, and PILS5 are suggested to transport auxin from the cytosol to the ER (Mravec et al., 2009; Wabnik et al., 2011). PILS2 and PILS5 are proposed to regulate auxin metabolism and signaling by increasing IAA conjugates and simultaneously decreasing nuclear auxin signaling, presumably by confining IAA in the ER (Barbez et al., 2012), whereas the pollen-specific PIN8 decreases IAA ER-compartmentation antagonistically (Dal Bosco et al., 2012; Ding et al., 2012). Therefore, these ER-localized auxin carriers affect auxin conjugation and link IAA transport to metabolism and signaling (Barbez and Kleine-Vehn, 2013; Kriechbaumer et al., 2015). In addition, the auxin-deconjugation, ILL2, IAR3, and ILR1, have been shown to localize in the ER where they are likely to produce free IAA by amidohydrolyzing IAA–amino acid conjugates (Ludwig-Müller, 2011; Sanchez Carranza et al., 2016). Considering the above clues, it is speculated that auxin conjugation could happen in the ER (Kriechbaumer et al., 2015). It is predicted that the ER functions as the main conduit for nuclear auxin uptake (Sauer and Kleine-Vehn, 2019). Given all of that, the ER serves not only as a platform for auxin biosynthesis but also as an auxin deposit and cycling hub (Friml et al., 2003). The disordered ER homeostasis and functions in the *mag2-1 mal-1* double mutant might affect auxin deposition and cycling.

In *mag2-1* cells, protein abundance of the phosphatase PP2A, which works antagonistically with kinase PID to regulate PIN cycling and activity, was decreased (Figure 5A). Breaking of balance of two enzymes with opposite functions will definitely influence PIN homeostasis in the PM. As a result, the protein abundance of PIN-GFP and PIN:GUS was altered significantly (Figure 7) and subsequently affected auxin transport and response in the *mag2-1 mal-1* double mutant (Figure 6).

Endoplasmic reticulum and auxin homeostasis maintenance by MAG2/MAL-mediated vesicle trafficking is essential for auxin transport and plant development, especially under stress conditions. Our study unveiled a novel perspective of membrane trafficking regulatory role in auxin homeostasis.

## Data Availability Statement

The datasets presented in this study can be found in online repositories. The names of the repository/repositories and accession number(s) can be found in the article/Supplementary Material.

## Author Contributions

LL and J-KZ conceived the project. LL and XNZ designed the experiments. XM, XMZ, HZ, ZL, YLiu, and XNZ conducted the experiments. XM, YZ, SS, YLi, and RL conducted the proteomics data analysis. HZ and LT conducted the confocal observation. LJ contributed reagents, materials, and analytical platform. LL and XM wrote the manuscript. All authors commented on the manuscript and approved the submitted version.

## Conflict of Interest

The authors declare that the research was conducted in the absence of any commercial or financial relationships that could be construed as a potential conflict of interest.

## Publisher’s Note

All claims expressed in this article are solely those of the authors and do not necessarily represent those of their affiliated organizations, or those of the publisher, the editors and the reviewers. Any product that may be evaluated in this article, or claim that may be made by its manufacturer, is not guaranteed or endorsed by the publisher.

## Footnotes

1
www.omicshare.com/tools

2
http://www.geneontology.org/

## Supplementary Material

Supplementary Table 1 Proteomic analysis of microsomal fraction of the *mag2-1 mal* double mutant.

Supplementary Table 2 Gene Ontology (GO) enrichment analysis of differently accumulated proteins (DAPs).

Supplementary Table 3 Vesicle trafficking-related DAPs.

Supplementary Table 4 Auxin-related DAPs.

Supplementary Table 5 Primers and probes used in this study.
