# Supplementary material for: MAG2 and MAL Regulate Vesicle Trafficking and Auxin Homeostasis With Functional Redundancy
Source: Front Plant Sci. 2022 Mar 16;13:849532. doi: 10.3389/fpls.2022.849532 (PMC8966843; doi:10.3389/fpls.2022.849532)
Supplement: Supplementary file 6 [file Data_Sheet_1.PDF]

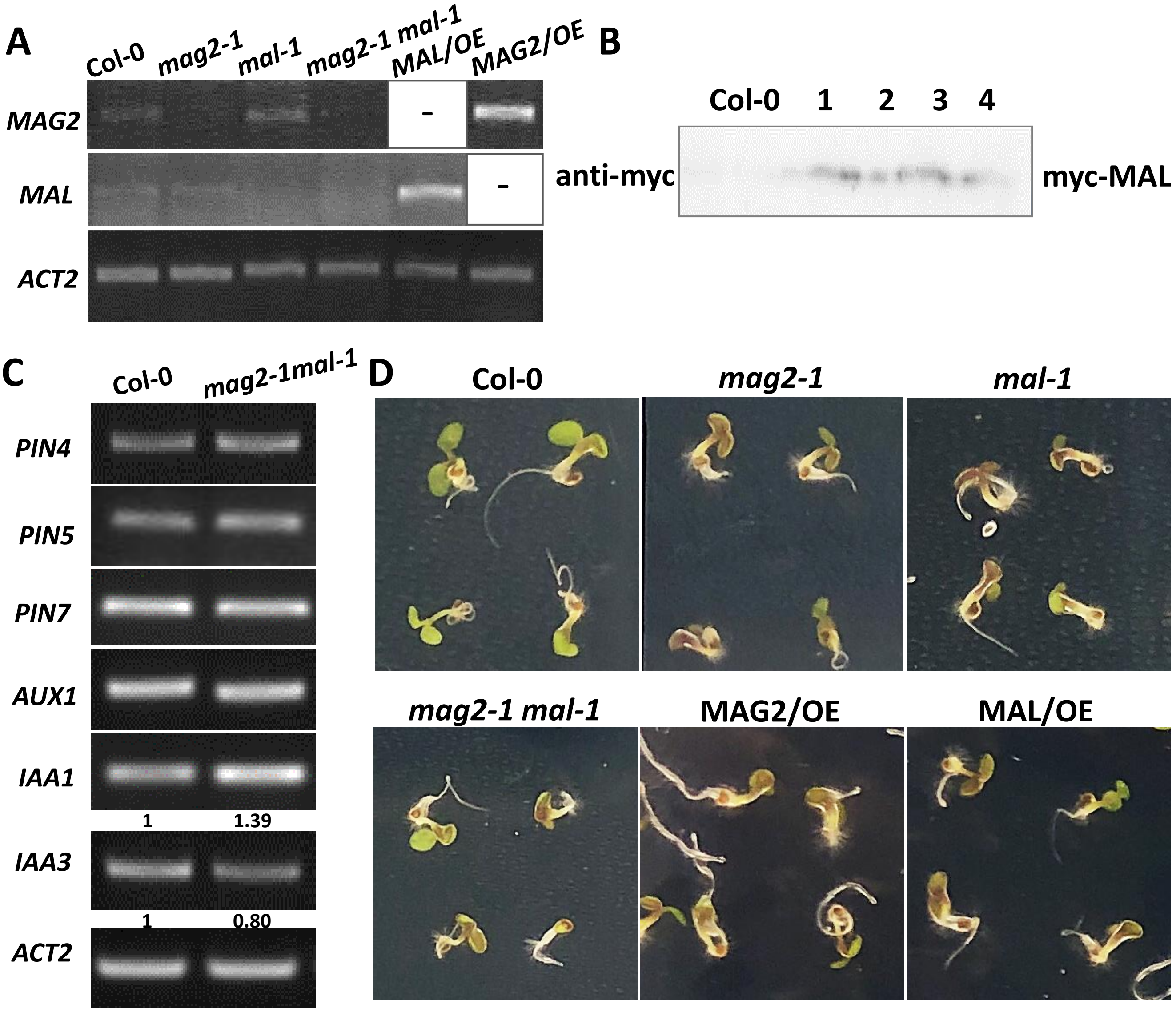

Supplementary FIGURE 1 | MAG2 and MAL affect plant development and stress response. (A) RT-PCR determination of *MAL* and *MAG2* expression levels in the mutants and overexpression lines. - : not performed. **(B)** Immunoblot detection of myc-MAL in *MAL*/OE lines. **(C)** RT-PCR detection of expression of auxin-related genes. Statistics of relative band concentration (presents expression level) is shown in number below the bands (*IAAs*/*ACT2*, measured by ImageJ). **(D)** Six-day-old seedlings germinated on 1/2 MS medium accumulated higher anthocyanin.
